# Effects of Hot Air Treatments on Postharvest Storage of Newhall Navel Orange

**DOI:** 10.3390/plants9020170

**Published:** 2020-02-01

**Authors:** Chunpeng Wan, İbrahim Kahramanoğlu, Jinyin Chen, Zengyu Gan, Chuying Chen

**Affiliations:** 1 Jiangxi Key Laboratory for Postharvest Technology and Nondestructive Testing of Fruits & Vegetables/Collaborative Innovation Center of Postharvest Key Technology and Quality Safety of Fruits & Vegetables in Jiangxi Province, College of Agronomy, Jiangxi Agricultural University, Nanchang 330045, China; chunpengwan@jxau.edu.cn (C.W.); ganzy@jxau.edu.cn (Z.G.); 2 European University of Lefke, Faculty of Agricultural Sciences and Technologies, Gemikonagi, via Mersin 10, 99780 Northern Cyprus, Turkey; ikahramanoglu@eul.edu.tr; 3 Pingxiang University, Pingxiang 337055, China

**Keywords:** hot air flowing, fruit decay, biochemical changes, malondialdehyde (MDA) content, enzyme activities

## Abstract

The effects of hot air flow (HAF) treatment on the postharvest storage of ‘Newhall’ navel oranges were investigated in this study. Studies were conducted with two separate sections. First of all, the effects of HAF at 37 °C for 36 h, for 48 h, and for 60 h were tested on fruit decay and weight loss. Thus, the optimal treatment was found as HAF at 37 °C for 48 h based on the fruit decay percentage and weight loss, and further studies were carried out with this treatment. The HAF-treated and control fruits were flowed at 37 °C and 20 °C with relative humidity (RH) of 85–95% for 48 h, respectively. After flowing, fruits of both treatments were individually film-packed, precooled (10–12 °C, 12 h), and stored (6 ± 0.5 °C and 85–90% relative humidity) for 120 days. Regular (0, 15, 30, 45, 60, 90, and 120 days) measurements were carried out for analyzing total soluble solid (TSS) content, titratable acid (TA) content, vitamin C (VC) content, total sugar content, respiration rate, malondialdehyde (MDA) content, and protective enzyme activities. The results indicated that HAF treatment significantly inhibited the MDA content and respiration rate of navel orange fruits after 45 d storage. The superoxide dismutase (SOD) and peroxidase (POD) enzyme activities were enhanced after 60 d storage, while polyphenol oxidase (PPO) enzyme activities were enhanced throughout the storage period. Results suggested that the SOD and POD activities are highly related with respiratory activities and could be enhanced with hot air flow. Meanwhile, HAF treatment maintained high content of TSS, total sugar, TA, and VC.

## 1. Introduction

Citrus is one of the most important fruit crops in the world, and best suited to grow in Mediterranean countries, China, and Brazil. Among the citrus species, the navel orange (*Citrus sinensis* L., Osbeck) is one of the most favored fruits for consumers due to its unique flavor, high nutritional contents, and bioactive compounds [1]. The most important postharvest problems for the storage of navel oranges are reported to be weight loss, chilling injury, and decay incidence (caused by *Penicillium italicum* and *P. digitatum*) [2]. Among these three problems, pathogens are classified as a large cause of postharvest losses [3]. The postharvest control of pathogens mostly relies on the use of synthetic agro-chemicals, which also had been reported to be the primary preference of the farmers [4]. However, the global pressure from consumers about the use of synthetic chemicals encourages scientific studies to develop eco-friendly and safer alternatives to fulfill consumers’ demands and to reduce postharvest losses [5]. Edible and medicinal plants, biological methods, and edible coatings were used for the control of citrus postharvest diseases [6,7,8]. Low temperatures are highly effective in reducing weight loss (caused by respiration) and decay incidence; however, citrus fruits are very sensitive to low temperatures [9]. Suggested temperatures for orange storage are between 5 and 8 °C [10]. 

Exposure to low temperatures for longer duration damages the cell membranes due to the production of reactive oxygen species (ROS) [11]. The two most important, safe, and novel methods are the use of edible coatings developed from essential oils, plant extracts, or other biomaterials [12,13,14,15] and preconditioning with heat treatment [16,17]. The activities of two important enzymes, peroxidase (POD) and superoxide dismutase (SOD), are known to alleviate lipid peroxidation [18,19], and activities of the polyphenoloxidase (PPO) enzyme improves tissues’ resistance to diseases in plants and fruits [20]. Furthermore, preconditioning with heat treatment such as hot dip water, hot forced air, or vapor heat is known to significantly influence the activities of those enzymes [21,22,23]. However, most of the previous studies have been conducted with hot water treatment [16,17,21,22,23,24,25,26,27,28,29,30,31,32,33,34,35] and very few research exists [25,36,37,38,39] for hot air use. Furthermore, early studies with hot water and hot air treatments on blood oranges reported adverse effects for hot air on the quality characteristics of the fruits [40]. Thus, the use of hot air for the protection of the postharvest storage quality of oranges is in question and needs to be studied further. Moreover, according to the authors’ knowledge, effects of hot air treatments on the postharvest storage quality of Newhall navel orange fruits had not been tested before. Therefore, in line with this information, the present study aimed to investigate the effects of hot air flow (HAF) treatment on the postharvest storage quality of Newhall navel oranges. Effects of HAF on the weight loss, fruit decay, and some biochemical characteristics were also investigated in the present study. 

## 2. Materials and Methods 

### 2.1. Materials

The fruit materials of the present study were Newhall navel oranges (*Citrus sinensis* L. Osbeck cv. Newhall). The present study was conducted in 2012 (preliminary studies) and 2013 (main studies). Fruits were harvested at commercial ripening from an orchard situated in the southeast of Ganzhou city (Jiangxi, China) in November 2012 and November 2013. The fruits were picked on the basis of fruit size (240–270 g) and uniform color (citrus color index, 3.5–4.8). Fruits with any mechanical injury, blemish, or diseases were discarded and uniform fruits were used in the present study.

### 2.2. Preliminary Studies 

A preliminary study was conducted to evaluate the effects of hot air flow (HAF) on the weight loss and decay percentage, and to determine the optimal treatment for the main studies. An initial experiment was performed with the following treatments: (1) no treatment as the control, (2) HAF at 37 °C for 36 h, (3) HAF at 37 °C for 48 h, and (3) HAF at 37 °C for 60 h. The previous report of Schirra et al. [40] carried out with blood oranges was used as a reference for the determination of the HAF temperature. Similarly, Lafuente et al. [41] used 37 °C in their studies for ‘Fortuna’ mandarins after our study. Overall, 240 uniform fruits (80 × 3 replications) were selected for each treatment in the preliminary studies in November 2012. Before storing the fruits, the initial weight of each fruit was measured with a digital scale and noted for further calculations. This data was then used together with the final weight of the fruits to calculate the weight loss (%) of each fruit. Fruits were then treated with the above-mentioned treatments. Next, all fruits were individually film-packaged, precooled (at 10–12 °C, 24 h), and finally stored at 6 ± 0.5 °C, and 85–90% relative humidity (RH) for 120 days. The decay percentage and weight loss for each treatment were recorded 120 d after storage. 

### 2.3. Decay Percentage and Weight Loss Determination for Preliminary Studies 

At the end of the storage period (120 d), all fruits were visually checked carefully one by one, and the fruits having more than 2% decay were noted as rotted. Thus, the percentage of fruit decay was calculated for each replication and then for each treatment. Weight loss determination was assessed with 20 fruits (randomly selected out of 80 fruits) from each replication. At the end of the storage period, the final weight of each fruit was measured and used with the previously measured initial weights for the calculation of the weight loss (%). 

### 2.4. Main Studies 

Preliminary experiments showed that the HAF at 37 °C for 48 h provides higher performance on the prevention of weight loss and fruit decay. Thus, the main studies were carried out with this treatment. The washed and air-dried fruits were randomly divided into two lots. One lot (30 fruits) was sampled before the treatments to evaluate the initial harvest quality (0 day), and the other lot (360 fruits) was randomly divided into two groups of 180 fruits each. Thus, 180 fruits were flowed at 37 °C and 20 °C with relative humidity (RH) of 85–95% for 48 h (60 × 3 replications), and another 180 fruits, placed at room temperature for 48 h, served as the controls (60 × 3 replications). Both HAF-treated and control fruits were individually film-packed (18 cm × 15 cm, Lingqu fresh packaging products Co. Ltd., Guilin, China) and precooled (at 10–12 °C, 12 h). Finally, all fruits were stored at 6 ± 0.5 °C and 85–90% relative humidity (RH) for 120 days. A total of 10 fruits from the HAF-treated and control groups were taken out at intervals of 15, 30, 45, 60, 90, and 120 d for analyzing the total soluble solid (TSS) content, titratable acidity (TA) content, vitamin C (VC: ascorbic acid) content, total sugar content, respiration rate, malondialdehyde (MDA) content, and protective enzyme activities. 

### 2.5. Biochemical Quality Analysis 

#### 2.5.1. TSS and Total Sugar

At each of the sampling points (0, 15, 30, 45, 60, 90, and 120 days), 10 fruits from each treatment were taken out and total soluble solid (TSS) and total sugar contents of the fruits were assessed individually (separately for each fruit). Juice of the fruit samples was extracted and TSS was measured by using an RA-250WE Brix Meter (Atago, Tokyo, Japan) and expressed as percentage. The total sugar content was then determined with the anthrone colorimetric method described by AOAC (Association of Official Analytical Chemists) [42] with some modifications. An amount of 5.0 g of juice samples was blended in 100 mL of distilled water and heated in boiling water for 30 min. After cooling, the above solution was added to 500 mL with distilled water. Then, 2.0 mL of the solution samples was mixed uniformly with 0.5 mL of anthrone reagent (1.0 g of anthrone dissolved in 50 mL of ethyl acetate) and 5.0 mL of concentrated sulphuric acid, incubated in boiling water for 5 min, cooled in ice water, and then determined at 630 nm. The total sugar content was calculated using a standard curve made by glucose, and expressed as percentage (%) on a fresh weight basis.

#### 2.5.2. TA and VC

The standard method described by AOAC [42] was used to assess TA and VC by titrating the samples with 0.1 M NaOH to an endpoint of pH 8.1 and 2,6-dichlorophenol indophenol. The contents of TA and VC were expressed as percentage of citric acid on a fresh weight basis and mg/100 g fresh weight, respectively. 

#### 2.5.3. Respiration Rate

The respiration rate was determined based on a method described by a previous study [13]. Respiration rate was measured by CO_2_ production using a GHX-3051H infrared CO_2_ fruit breathing apparatus (Jingmi Scientific LLC., Shanghai, China) and expressed as mg kg^−1^ h^−1^. 

#### 2.5.4. MDA Content

The malondialdehyde (MDA) contents of the HAF-treated and untreated control groups were measured according to the method of Hodges et al. [43]. For this analysis, pericarp tissues of 10 fruits (for each treatment) were ground individually in a MM 400 frozen grinder (Retsch GmbH., Arzberg, Germany), and 2.0 g of powder was homogenized in 25 mL of ice-cold 50 mM phosphate buffer (pH 7.8) containing 1 mM ethylenediaminetetraacetic acid (EDTA) and 2% (*w*/*v*) polyvinylpyrrolidone (PVP). Thereafter, the homogenized samples were centrifuged at 12,000 *g* (5804R, Eppendorf, Hamburg, Germany) for 20 min at 4 °C. Next, 2 mL of the supernatant was mixed with 2 mL of 0.5% (*w*/*v*) thiobarbituric acid (TBA) and further incubated in boiling water for 30 min. Samples were then cooled and then centrifuged at 6000g (5804R, Eppendorf) for 10 min. Afterwards, the absorbance of supernatant was measured at three different wavelengths (450, 532, and 600 nm) using an M5 Multiscan Spectrum microplate reader (Molecular Devices Corporation, Sunnyvale, California, USA). The MDA content was then calculated according to the formula (6.452 × (*A*_532_ − *A*_600_) − 0.559 × *A*_450_) and expressed as mmol g^−1^ frozen weight (FW).

#### 2.5.5. Protective Enzymes Activities

The analyses of three different protective enzymes were performed separately for 10 fruits from each treatment. First of all, aliquots of fresh powder (2.0 g) were homogenized with various ice-cold extraction buffers to prepare extracts for assay of the following protective enzymes: 10 mL of 50 mM ice-cold phosphate buffer (pH 7.8) containing 1 mM EDTA, 5 mM dithiothreitol (DTT), and 2% (*w*/*v*) PVP for superoxide dismutase (SOD, EC 1.15.1.1); 8 mL of 100 mM ice-cold acetate buffer (pH 5.5) containing 1 mM polyethylene glycol (PEG), 4% (*w*/*v*) PVP, and 1% (*w*/*v*) Triton X-100 for peroxidase (POD, EC 1.11.1.7) and polyphenol oxidase (PPO, EC 1.10.3.1). All homogenates were centrifuged at 12,000 *g* (5804R, Eppendorf) for 30 min at 4 °C and then the supernatants were collected for the enzyme activity assays.

Superoxide dismutase (SOD) activity was assayed by measuring its ability to inhibit the photoreduction of nitroblue tetrazolium (NBT) according to the method of Ballester et al. [18] with slight modifications. The reaction mixture consisted of 1.5 mL phosphate-buffered saline (PBS, 50 mM), 0.3 mL methionine (Met, 130 mM), 0.3 mL NBT (0.75 mM), 0.3 mL ethylenediaminetetraacetic disodium dihydrate (EDTA-Na_2_, 0.1 mM), 0.3 mL riboflavin (20 µM), 0.1 mL enzyme extract, and 0.5 mL distilled water in a total volume of 3.3 mL. The mixtures were illuminated by light (4000 Lx) for 20 min at 28 °C and the absorbance was then determined at 560 nm (Shimadzu UV-2600, Tokyo, Japan). One unit of SOD activity was defined as the amount of enzyme that would inhibit 50% of NBT photoreduction and expressed as U min^−1^ g^−1^.

Peroxidase (POD) activity was based on the measurement of guaiacol oxidation at 470 nm in the presence of H_2_O_2_. The collected supernatant (100 μL) was mixed with 3.0 mL of 25 mM guaiacol and 200 μL of 50 mM H_2_O_2_. Oxidation of guaiacol was determined at 470 nm for 3 min. One unit of POD activity was defined as an increment of 0.01 in absorbance per minute at 470 nm (Shimadzu UV-2600, Tokyo, Japan) and expressed as U min^−1^ g^−1^.

Polyphenoloxidase (PPO) activity was based on the measurement of catechol oxidation at 420 nm. The collected supernatant (200 μL) was mixed with 4.0 mL of 50 mM acetate buffer (pH 5.5) and 1.0 mL of 50 mM catechol. Oxidation of catechol was determined at 420 nm for 5 min. One unit of PPO activity was defined as an increment of 0.01 in absorbance per hour at 420 nm (Shimadzu UV-2600, Tokyo, Japan) and expressed as U h^−1^ g^−1^.

### 2.6. Statistical Analysis

Data from the preliminary studies was subjected to analysis of variance to determine if there were any significant differences among the treatments. Thereafter, if there were any significant differences, statistical separation of the means was performed with the Duncan’s multiple range test (*P* < 0.05). The data was then subjected to the independent samples *t*-test at *P* < 0.05 for the comparison of the biochemical parameters of the main studies. All statistical analysis was performed with the SPSS software (Version 17.0, SPSS Inc., Chicago, IL, USA).

## 3. Results

### 3.1. Hot Air Flow (HAF) Treatments on Decay Percentage and Weight Loss of Navel Orange

**Figure 1:**
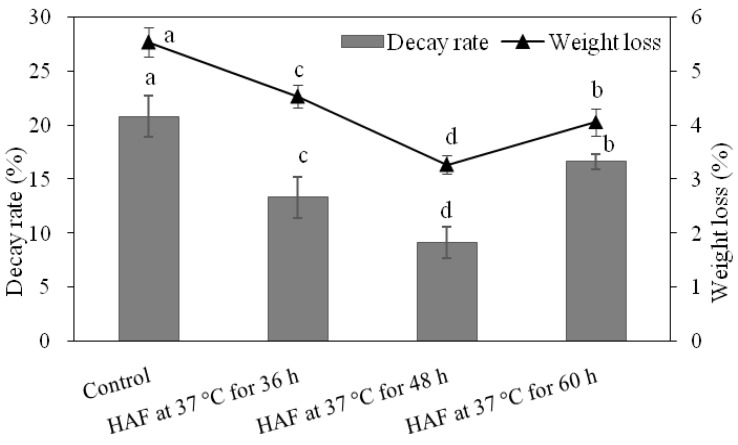
Effect of different HAF treatments on decay percentage and weight loss of navel orange fruit stored at 6 °C for 120 days. Values are the mean ± S.E. (n = 3).

The two most important parameters of the postharvest quality determination are the weight loss and decay percentage, which are also used for the determination of the success of the postharvest preservatives. Results of the preliminary studies about the effects of hot air flow (HAF) on the weight loss and decay percentage of the Newhall navel orange are summarized in Figure 1. It is clear from the figure that the highest influence on both the decay percentage and weight loss was obtained from HAF at 37 °C for 48 h. Results also showed that increasing the duration of the HAF increased the efficacy of the treatment for a period, and then it caused a reduction in the efficacy. According to our results, the highest weight loss was registered at the end of cold storage (120 days) in control fruits. The weight loss of the control fruits was found to be 5.54% and was followed by the HAF at 37 °C for 36 h treatment with 4.53%. The highest success or, in other words, the least weight loss was measured from the HAF at 37 °C for 48 h treatment which was only 3.28%. Similar results were obtained for decay percentage. At the end of the storage duration, the highest fruit decay was noted from control fruits with 20.83%, where the decay percentage of the fruits treated with HAF at 37 °C for 48 h was only 9.17%.

### 3.2. Effects of HAF on Some of the Postharvest Fruit Quality Parameters

**Figure 2:**
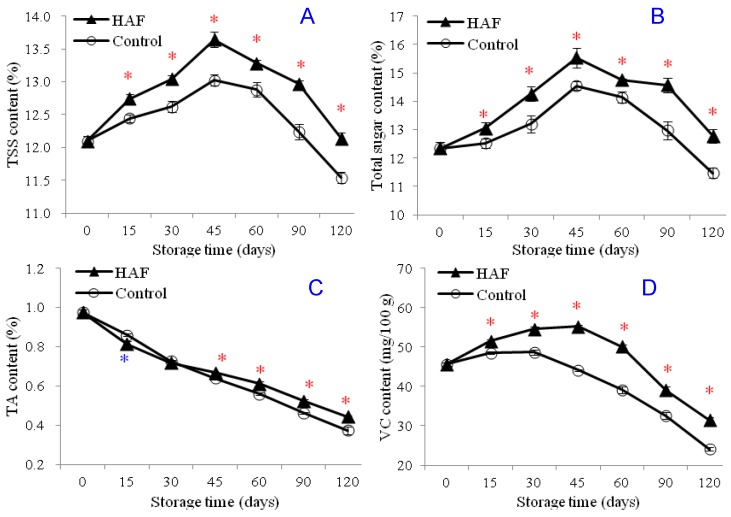
Effects of HAF treatment on the contents of TSS (**A**), total sugar (**B**), TA (**C**), and VC (**D**) of navel orange fruits during 120 days of cold storage at 6 °C. Each value was presented as the mean ± standard error (SE) of three replicates. The symbols * and * represent significantly higher and lower values, respectively, for the HAF-treated fruits than for the control fruits. The significant difference was determined according to the independent samples *t*-test (*P* < 0.05) on each storage day.

Total soluble solids (TSS), total sugar, titratable acidity (TA), and Vitamin C (VC) are among the most important indicators of the postharvest storage quality and important determinants for the consumers’ demand. The TSS/TA ratio is an important indicator of the product’s taste. Present results showed that the TSS content of the Newhall navel orange fruits had an increasing trend during the initial periods of the storage and after about 45 days of storage, it showed a decreasing trend which came to a lower point than the initial point for the control fruits (Figure 2A). The initial TSS content of the fruits was 12.10% just after the harvest (0 days of storage) and it was measured as 11.54% for the control fruits at the end of the storage period (120 days of storage). At the end of the storage period, the TSS content of the HAF-treated fruits was 12.14%, which was slightly above the initial content. According to the results, the TSS content of the HAF-treated fruits was higher than that of the control fruits for the whole duration of cold storage. The changes in the total sugar content of the HAF-treated and control fruits were found to be similar to the variation in the TSS content of the fruits. It was found to increase during the initial phases of the storage and had a downward trend after 45 days of storage. Similarly to TSS content, total sugar content of the HAF-treated fruits was higher than that of the control fruits. Contrary to the TSS and total sugar contents, the TA content of the fruits showed a decreasing trend for the duration of cold storage. During the initial periods of the storage (until 45 days of storage), the TA content of the control was slightly higher than or significantly similar with that of the HAF-treated fruits. Afterwards, the TA content of the HAF-treated fruits was found to be significantly higher than that of the control group (Figure 2C). Finally, the VC content of the fruits was found to have a slight increase during the initial periods of the storage duration and then it was noted to have a rapid decrease (Figure 2D). Results of the present study showed that the HAF treatment significantly protects the VC content and delays its decrease. 

### 3.3. Effect of HAF on Respiration Rate and MDA Content of Navel Orange 

**Figure 3:**
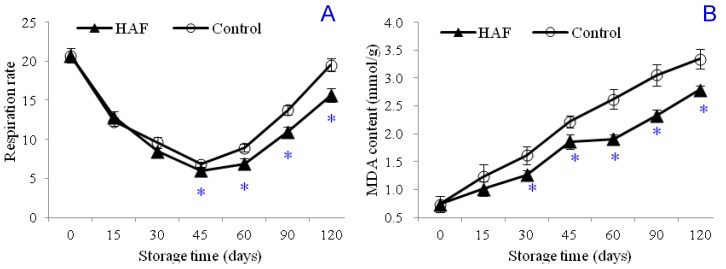
Effects of HAF treatment on respiration rate (**A**) and MDA content (**B**) of navel orange fruits during 120 days of cold storage at 6 °C. Each value was presented as the mean ± standard error (SE) of three replicates. The symbol * represents significantly lower values for the HAF-treated fruits than for the control fruits. The significant difference was determined according to the independent samples *t*-test (*P* < 0.05) on each storage day.

Respiration significantly influences the postharvest storage quality of fresh produce. Fresh products lose weight and quality as they respire by breaking down the stored carbohydrates into end products (H_2_O, CO_2_, and energy). Furthermore, the speed of respiration is highly important for the protection against postharvest losses. Results of the present study showed that the respiration rate of the navel orange fruits decreased during the initial periods (initial 45 days of storage) of cold storage and then it showed an increasing trend (Figure 3A). According to the results of the current work, HAF treatment is effective in keeping the respiration rate lower after the first 45 days of storage. Respiration rate was measured as CO_2_ production of the fruits in the present study, and the initial respiration rate was calculated as 20.72 mg kg^−1^ h^−1^. The production of CO_2_ decreased during the storage duration and was measured as 5.98 mg kg^−1^ h^−1^ for the HAF-treated fruits and as 6.82 mg kg^−1^ h^−1^ for the control fruits 45 days after storage. At the end of the cold storage (120 days after storage), the CO_2_ production of the control fruits was measured as 19.49 mg kg^−1^ h^−1^, which was close to the initial respiration rate, and was measured as 15.61 mg kg^−1^ h^−1^ for the HAF-treated fruits, which was significantly lower than for the control fruits. Results of the present study, on the other hand, showed that the HAF treatment was effective in reducing the malondialdehyde (MDA) content of the fruits. MDA is the end product of the lipid peroxidation, which suggests that the HAF treatment reduces the lipid peroxidation in the cells of stored fruits. The MDA content of the fruit samples was 0.73 mmol g^−1^ at the beginning of the experiments and was found to increase to 3.34 mmol g^−1^ at the end of the storage duration (Figure 3B). This was about 3.57-fold higher than the initial MDA content. Here, the HAF treatment was found to be effective in reducing the MDA content, which was measured as 2.79 mmol g^−1^ for the HAF-treated fruits. 

### 3.4. Effects of HAF on Enzyme Activities of SOD, POD, and PPO of Navel Orange

**Figure 4:**
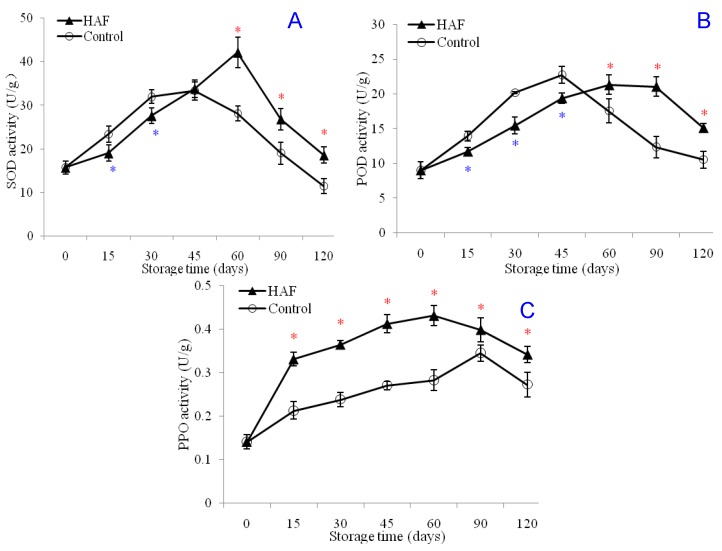
Effects of HAF on enzyme activities of SOD (**A**), POD (**B**), and PPO (**C**) of navel orange fruits during 120 days of cold storage at 6 °C. Each value was presented as the mean ± standard error (SE) of three replicates. The symbols * and * represent significantly higher and lower values, respectively, for the HAF-treated fruits than for the control fruits. The significant difference was determined according to the independent samples *t*-test (*P* < 0.05) on each storage day.

Peroxidase (POD) and superoxide dismutase (SOD) enzymes are known to alleviate lipid peroxidation in fruits [18,19]. Results of the present study showed that the hot air flow (HAF) treatment significantly influenced the activities of these highly important enzymes (i.e., POD and SOD). According to the obtained results, Both SOD and POD enzymes showed an increasing trend during the initial periods of cold storage (until 45 d), and then both SOD and POD showed a decreasing trend (Figure 4A,B). During this initial period, fruits treated with HAF were found to have lower SOD and POD activities as compared with control. Moreover, the increasing trend of these enzymatic activities was found to continue until 60 days, instead of 45. Thereafter, the SOD and POD activities of HAF-treated fruits were found to be higher than the control group. Results suggested that the HAF treatment had significant influence on the POD and SOD enzymes. The SOD activity was 15.73 U g^−1^ at the beginning of the storage period and reached peak level (33.36 U g^−1^) at 45 days of storage for the control group and decreased to 11.46 U g^−1^ at 120 days of storage. The SOD activity of the HAF-treated fruits was significantly similar to the SOD activity of the control group at the 45th day of storage (33.84 U g^−1^). The highest SOD activity was found to be 42.15 U g^−1^ for the HAF-treated control fruits at the 60th day of storage, and it showed a decreasing trend after 60 days of storage. The initial, peak, and final activities of POD were found to be 9.01 U g^−1^, 21.32 U g^−1^, and 15.14 U g^−1^, respectively, for HAF-treated navel orange fruits. Polyphenoloxidase (PPO) enzyme was previously reported to improve tissues’ resistance to diseases [20]. According to the results of the present study, the PPO activity of navel oranges increased during the storage period until 60–90 days of storage and then it showed a decreasing trend (Figure 4C). Results showed that the HAF-treated fruits had significantly higher PPO activities during the whole storage period. However, the PPO activities of the HAF-treated fruits also showed a decreasing trend after 90 days of storage. The PPO activity of the navel orange fruits was measured as 0.14 U g^−1^ and it reached a maximum level (0.40 U g^−1^) in 60 days of storage for the HAF-treated fruits. Results for the PPO activity are in agreement with the preliminary studies of this research, where the decay rates of the HAF-treated fruits were lower than those of the control fruits. 

### 3.5. Relationships between the Observed Parameters

Relationships between the observed parameters are given in Table 1. Results showed that most of the parameters had significant correlation for the HAF-treated fruits, while the correlation was insignificant or weak for the control fruits. The PPO, POD, and SOD enzymatic activities were found to have high correlation for the HAF-treated fruits. This correlation was also significant for the control group only for POD vs. SOD and it was not significant for PPO vs. POD and PPO vs. SOD. As a sign of the oxidation, MDA content was found to have a high correlation with the PPO activity at the control group. All of the enzyme activities were also found to have high correlation with the respiration rate and total sugar.

## 4. Discussion

Numerous studies have been conducted about the postharvest influences of hot water treatment [16,17,21,22,23,24,25,26,27,28,29,30,31,32,33,34,35]; however, very few studies [25,36,37,38,39] have been carried out with hot air. Results of the present study clearly showed that the hot air flow (HAF) treatment significantly influences the weight loss and decay percentage of the navel orange fruits. However, results also showed that the duration of exposure of fruits to HAF is highly important and significantly influences the positive impact of HAF. For the present study, the highest influence on both the decay percentage and weight loss was obtained from HAF at 37 °C for 48 h. In one of the above-mentioned previous studies, Belovic et al. [37] reported that the hot air and ultraviolet (UV) radiation have significant influence on the sensory quality of tomatoes and might be used to extend shelf life of tomato fruits due to delayed ripening. In the other study, Erkan et al. [25] noted that heat treatments, either hot water dipping or curing with hot air, are effective for reducing fruit decay in ‘Clementine’ mandarins. Hot air treatment was also reported to increase the antifungal compounds of fruits, which in turn helps to control postharvest pathogens [44]. In a different but closely linked study, Wang et al. [45] reported that hot water treatment increases SOD activities which in turn results in a decrease in *Rhizopus* rot in peaches. Higher activity of SOD and reduced decay percentage were then noted for sweet cherry fruit [36]. These studies are in agreement with the present study, where the preliminary studies showed that HAF significantly reduces decay percentage and the main studies showed that HAF treatment increases the activities of SOD. PPO and POD were previously reported to be responsible for the oxidation of phenolics in plant cells into antimicrobial quinones and protecting plants from pathogens [46]. A similar relationship between decay percentage and PPO and POD activities was noted for muskmelon fruits. Hot water, not hot air, was noted to enhance the activities of PPO and POD in muskmelon and reduce decay percentage during storage [29]. Antifungal activities of hot water treatment were previously reported for different crops (i.e., banana [30], squash [47], and apples [32]). A similar effect was previously noted by Palou et al. [24] on the ‘Clementine’ mandarins where hot water treatment was found to reduce the incidence of green and blue molds. In a different study by Plaza et al. [48], it was reported that heat curing of oranges at 33 °C for 65 h resulted in a significant reduction in green mold. This result is also in agreement with the results of the present study, which shows that HAF is effective for controlling postharvest fungal pathogens, but the temperature and curing duration are highly important. In another similar study, Liu et al. [49] tested the effects of hot air (38 °C for 36 h) and *Pichia guilliermondii* alone or in combination against anthracnose rot in loquat fruits. They noted that the hot-air-treated fruits had lower SOD activities during the early stages of storage and the enzymatic activities increased later. They also reported that the effects of hot air on the mycelia growth increased when it was combined with *P. guilliermondii*. Antifungal activities of hot air treatment were previously reported for Chinese bayberry (48 °C for 3 h by [44]), cherry tomato (38 °C for 12 h by [50]), and strawberry (45 °C for 3 h by [39]). 

The main studies of the present study showed that HAF treatment not only prevents weight loss and fruit decay, but also significantly improves total soluble solids (TSS), total sugar, titratable acidity (TA), and Vitamin C (VC) contents of navel orange fruits during cold storage. Similar results were previously reported by Erkan et al. [25] who noted that hot air treatment delays the reduction of TSS and VC. However, they noted that the hot air reduces TA content. This could be due to the differences in the temperature and duration combinations for HAF treatment. In another study with ‘Satsuma’ mandarins, it was noted that hot water treatment prevents the reduction in TSS and TA; however, the efficacy varies among the different temperatures. Researchers noted that the optimum temperature is 50 °C for 3 min and higher temperatures decrease the efficacy of the treatment. They also reported that hot water treatment slightly increases VC content but no significant difference was reported with untreated control fruits [28]. The fruits were heated until the core temperature reached 44 °C and kept at that temperature for 100 min. Researchers reported that for fruits stored for 3 weeks at 5 °C followed by shipping and marketing simulations at 20 °C and 13 °C for 4 and 3 days, respectively, although the HTFA treatment increased the SSC/TA ratio, it also caused an important damage in product flavor. In a previously published article, Gao et al. [51] studied the effects of hot air treatment (HAT 40 °C, 48 h) on the citric acid contents of ponkan fruits. Researchers noted that the citric acid concentration (consistent with TA) decreased during the storage duration while the HAT promoted citric acid degradation. They also noted that the hot treatment enhanced the expression of some genes, which are related to degradation of citric acid. In a previous study with blood oranges, the effects of hot water dipping (50 °C for 3 min) and hot air treatment (37 °C for 48 h) were tested against chilling injury and fruit decay. In contrast to our studies, in the mentioned study, Schirra et al. [40] reported that the hot air treatment caused a significant decrease in quality attributes of the blood orange fruits. Vitamin C is an important marker for the products’ resistance to oxidative stress. Biosynthesis of VC was reported to enhance scavenging of reactive oxygen species (ROS) [52]. VC concentration was also reported to increase in tomato fruits treated with hot water [53]. In line with this information, Massot et al. [54] reported that heat stress might activate genes related with VC biosynthesis. The amount of VC at the chilled temperatures helps to prevent chilling injury by neutralizing free radicals [33]. Previous studies with different temperature and duration combinations on different produce showed positive effects on VC biosynthesis and decrease of ROS. It was previously reported that the postharvest treatments and the oxidative stress significantly influence the VC contents of the citrus fruits. Studies also suggested that the heat treatments improve VC retention in citrus fruits. Mditshwa et al. [38] in their review paper discussed the changes in VC content of citrus fruits after heat treatment and noted that the varying responses of the fruits could be hypothesized to be caused by different morphological and ultrastructural features of the flavedo. They also suggested that the gas permeability of the fruit peel has significant influence on VC variation. It was then reported that the internal oxygen leads oxidation of ascorbic acid and thus reduces the VC content. Our results support this knowledge, where the respiration rate of the fruits had a reverse relationship with the VC content. Thus, in line with the hypothesis of Mditshwa et al. [38], it can be concluded that hot air treatment redistributes the natural epicuticular wax on the fruit surface which forms a gas barrier and this might reduce oxidation of ascorbic acid. 

Respiration is the basic biochemical process in which the fruit cells convert carbohydrates into energy by combining oxygen and glucose. This process causes the stored sugar to decompose and the fruits to deteriorate. Results of the present study showed that the respiration rate of the fresh navel orange fruits decreased during the initial periods (initial 45 days) of cold storage and then it showed an increasing trend. It converted stored sugars into energy and increased weight loss. Thus, respiration is one of the crucial factors determining the postharvest quality and storability of fresh produce. Results also showed that HAF treatment is effective in keeping the respiration rate lower. Similar results were reported by Opio et al. [31] who noted that hot water treatment reduces the respiration rate and ethylene production of lime fruits during storage. Some other studies suggest that hot water treatment has no obvious effect on the respiration rate of citrus fruit [21]. These results are also supported in the present study, where the hot air treatment was found to have no effects on the respiration rate during the first 45 days of storage. Apart from the respiration rate, malondialdehyde (MDA) content is the other important factor determining the postharvest quality of fresh produce. It is considered a biomarker of oxidative damage [55] and a secondary end product of polyunsaturated fatty acid oxidation, and is a commonly used method for the determination of lipid peroxidation [56]. Results of the present study showed that the HAF treatment is effective in reducing the MDA content of the fruits. Similarly, Lafuente et al. [41] reported that heat conditioning at 37 °C for 3 days regulates the stress-related proteins in cold-stored ‘Fortuna’ mandarins. Heat conditioning is reported to induce the expression of genes that encode late embryogenesis-abundant (LEA) proteins and to reduce reactive oxygen species (ROS) formation which is highly associated with membrane damage. Endo et al. [34] also reported that hot-water-treated green mume (*Prunus mume*) fruits had lower MDA content (representing lower lipid peroxidation). 

Peroxidase (POD) and superoxide dismutase (SOD) enzymes are known to alleviate lipid peroxidation [18,19]. SOD is also an ever-present defensive enzyme and is known to protect tissues from the superoxide damage to anaerobic organisms [19]. Results of the present study showed that HAF is effective in increasing the activities of POD and SOD. Moreover, it was found that the activities of POD and SOD begin to increase after the respiration rate reaches its lowest peak. Results suggest that the increasing activities of POD and SOD are highly related with the protection of the postharvest quality of navel orange fruits. Similar results were previously reported by Yun et al. [21] for SOD and POD activities in citrus fruits. They noted that the SOD and POD activities were higher in the treated fruits in the first six days of storage and then decreased below those of control treatments and then increased again. The fluctuating activity of SOD and POD in this literature supports the results of the present study. On the other hand, HAF was previously reported to increase the activity of SOD and reduce the activities of POD [57]. In a previously conducted study with strawberry fruits, it was reported that the hot air (45 °C, 3.5 h) application before storage reduced decay caused by *Botrytis cinerea* by increasing the resistance mechanism. In that study, Peng et al. [58] noted that the hot air treatment increased the activities of SOD and CAT enzymes and expressed three defence-related genes (CAT, CCR-1 allele, and PLA6). POD is known to prevent cells from free radicals under stress [19,59], and the positive effects of HAF on POD activity is important for the protection of the postharvest quality of navel orange fruits. The other important enzyme in fruits is polyphenoloxidase (PPO), which is reported to improve tissues’ resistance to diseases [20]. Results of the present study showed that PPO activity of navel oranges increased during the storage period and HAF treatment significantly increased the enzymatic activities. As noted above, very few studies had been conducted about the postharvest effects of hot air and no information is available about its efficacy on PPO activity. However, the positive effect of hot water on the PPO activity was noted previously [29]. 

To sum up the results, the use of novel postharvest technologies, especially nonchemical procedures (i.e., essential oils [15]), is very important for ensuring sustainability in production and ensuring food safety. Among the nonchemical technologies, hot air treatment has some limitations when compared with hot water treatment; however, it has some advantages when compared with edible coatings, radio frequency, hyperbaric pressure, and ultraviolet radiation [60]. The main disadvantages of hot air treatment which prevent its commercial utilization are the expense of heating and difficulties in immobilizing large amounts of fruit. Further studies need to be focused on the elimination of those disadvantages.

## 5. Conclusions

The present study showed that the hot air flow (HAF) treatment has positive effects on the postharvest quality and life of Newhall navel orange fruits. Results suggested that the HAF duration is highly important for maximum prevention of weight loss and fruit decay, and the optimum HAF treatment for Newhall navel orange fruits is 37 °C for 48 h. Results also showed that the HAF treatment significantly improves the TSS, TA, total sugar, and VC convents of the navel oranges. Present results suggested that the HAF treatment significantly reduces respiration rate, delays fruit deterioration, and reduces the MDA content of the stored fruits, a sign of the reduction in oxidative damage and lipid peroxidation. The SOD and POD activities of the navel orange fruits were found to have an inverse relationship with the respiration rate of the fruits, and the HAF treatment was found to increase the activities of these enzymes after a critical period of storage duration. It was also found that the PPO activity of the HAF-treated fruits was significantly higher than the control group, as a sign of the increased resistance to fruit decay. 

## Figures and Tables

**Table 1:** Pearson correlation among the observed quality parameters of the HAF-treated and control fruits.

Parameters	TA	VC	TS	RR	MDA	SOD	POD	PPO
**TSS–HAF**	−0.196	0.664	0.958 **	−0.949 **	0.093	0.848 *	0.734	0.774 *
**TSS–Control**	0.223	0.634	0.952 **	−0.910 **	−0.215	0.939 **	0.853 *	0.115
**TA–HAF**		0.541	−0.377	0.407	−0.971 **	−0.343	−0.721	−0.724
**TA–Control**		0.855 *	0.003	0.089	−0.996 **	0.162	−0.059	−0.872 *
**VC–HAF**			0.469	−0.539	−0.649	0.442	0.017	0.159
**VC–Control**			0.407	−0.422	−0.868 *	0.635	0.433	−0.583
**TS–HAF**				−0.928 **	0.301	0.865 *	0.860 *	0.809 *
**TS–Control**				−0.876 **	0.013	0.868 *	0.836 *	0.292
**RR–HAF**					−0.267	−0.870 *	−0.798 *	−0.904 **
**RR–Control**					−0.078	−0.943 **	−0.936 **	−0.401
**MDA–HAF**						0.264	0.664	0.596
**MDA–Control**						−0.186	0.028	0.879 **
**SOD–HAF**							0.837 *	0.763 *
**SOD–Control**							0.957 **	0.128
**POD–HAF**								0.883 **
**POD–Control**								0.265

* represents significant correlation at the 0.05 level and ** used for the 0.01 level (two-tailed).
